# Yeast-encapsulated essential oils: a new perspective as an environmentally friendly larvicide

**DOI:** 10.1186/s13071-019-3870-4

**Published:** 2020-01-13

**Authors:** Michael J. Workman, Bruno Gomes, Ju-Lin Weng, Linnea K. Ista, Camila P. Jesus, Mariana R. David, Marcelo Ramalho-Ortigao, Fernando A. Genta, Scott K. Matthews, Ravi Durvasula, Ivy Hurwitz

**Affiliations:** 1 Center for Global Health, University of New Mexico Health Sciences Center, Albuquerque, NM USA; 2 Department of Chemical and Biological Engineering, University of New Mexico, Albuquerque, NM USA; 3 Laboratório de Bioquímica e Fisiologia de Insetos, Instituto Oswaldo Cruz (IOC-Fiocruz), Rio de Janeiro, Brazil; 4 Department of Preventive Medicine and Biostatistics, Uniformed Services University, Bethesda, MD USA; 5 Laboratório de Mosquitos Transmissores de Hematozoários, Instituto Oswaldo Cruz (IOC-Fiocruz), Rio de Janeiro, Brazil; 6 Instituto Nacional de Ciência e Tecnologia em Entomologia Molecular, Rio de Janeiro, Brazil; 7 Department of General Preventive Medicine, School of Medicine and Dentistry, University of Rochester, Rochester, NY USA; 8 Loyola University Stritch School of Medicine, Maywood, IL USA

**Keywords:** Larvicide, Essential oil, *Saccharomyces cerevisiae*, Encapsulation

## Abstract

**Background:**

Effective mosquito control approaches incorporate both adult and larval stages. For the latter, physical, biological, and chemical control have been used with varying results. Successful control of larvae has been demonstrated using larvicides including insect growth regulators, e.g. the organophosphate temephos, as well as various entomopathogenic microbial species. However, a variety of health and environmental issues are associated with some of these. Laboratory trials of essential oils (EO) have established the larvicidal activity of these substances, but there are currently no commercially available EO-based larvicides. Here we report on the development of a new approach to mosquito larval control using a novel, yeast-based delivery system for EO.

**Methods:**

Food-grade orange oil (OO) was encapsulated into yeast cells following an established protocol. To prevent environmental contamination, a proprietary washing strategy was developed to remove excess EO that is adsorbed to the cell exterior during the encapsulation process. The OO-loaded yeast particles were then characterized for OO loading, and tested for efficacy against *Aedes aegypti* larvae.

**Results:**

The composition of encapsulated OO extracted from the yeast microparticles was demonstrated not to differ from that of un-encapsulated EO when analyzed by high performance liquid chromatography. After lyophilization, the oil in the larvicide comprised 26–30 percentage weight (wt%), and is consistent with the 60–65% reduction in weight observed after the drying process. Quantitative bioassays carried with Liverpool and Rockefeller *Ae. aegypti* strains in three different laboratories presented LD_50_ of 5.1 (95% CI: 4.6–5.6) to 27.6 (95% CI: 26.4–28.8) mg/l, for L1 and L3/L4 mosquito larvae, respectively. LD_90_ ranged between 18.9 (95% CI: 16.4–21.7) mg/l (L1 larvae) to 76.7 (95% CI: 69.7–84.3) mg/l (L3/L4 larvae).

**Conclusions:**

The larvicide based on OO encapsulated in yeast was shown to be highly active (LD_50_ < 50 mg/l) against all larval stages of *Ae. aegypti*. These results demonstrate its potential for incorporation in an integrated approach to larval source management of *Ae. aegypti*. This novel approach can enable development of affordable control strategies that may have significant impact on global health.

## Background

Mosquito-borne diseases are a global threat to human health, implicated in over 1.2 million deaths annually [1]. Among these, those increasing at the fastest rate are the arboviruses dengue, chikungunya and Zika. In the absence of vaccines and effective treatment, public health responses have focused on preventing transmission by reducing vector populations. Unfortunately, these arboviruses are transmitted by the *Aedes aegypti* mosquito, a vector presenting formidable challenges to disease control. *Aedes aegypti* feeds during daylight hours, does not feed to repletion, and is primarily anthropophilic with preference for urban and exurban areas. Current approaches to control adult *Ae. aegypti* relying on insecticide spraying in urban areas pose risks to human and environmental health, and often select for mosquito resistance, making them ineffective and unsustainable [2].

Mosquito control using larvicides offers a potential solution to regional patterns of mosquito-borne disease transmission. Compared to sprayed insecticides targeting adult mosquitoes, larvicides are simpler and safer to implement. High kill rates can be achieved as larvae are confined to aquatic environments and are unable to evade control measures. However, synthetic larvicides currently in use, including carbamates, pyrethroids, organophosphates and organochlorides, exhibit one or more of four disadvantages: (i) toxicity to humans and other non-target species; (ii) degradation of the aquatic environment; (iii) high annual cost; and (iv) vulnerability to the evolution of target resistance. Alternatively, an ideal larvicide would have the following attributes: (i) non-toxic to humans and other non-target species; (ii) able to offer high kill rates over a short period of time; (iii) ecologically appropriate in application, with minimal alteration of the aquatic breeding environment; (iv) capable of retarding and/or responding to evolution of target species resistance; (v) inexpensive, and readily scalable; and (vi) designed to provide protection to the most vulnerable populations in areas with the highest rates of mosquito-borne diseases.

Essential oils (EO) are volatile oils with strong aromatic components imparting distinctive flavors or scents, and have a long history of commercial use, ranging from pharmaceuticals to flavor additives for foods [3]. While recognized as non-toxic to humans, these secondary metabolites are produced by plants for protection against pathogenic microorganisms and predator insects. EOs are highly complex natural mixtures, often containing between 20–60 components. Of these, 2 or 3 components are present at distinctly high concentrations and, generally, it is these components that determine biological activity of the EO. Terpenes or terpenoids are common primary constituents of EOs, as are aromatic or aliphatic molecules.

In recent years, larvicidal activities of EOs from a number of plants [4, 5], including *Citrus* essential oils [6–9], *Cryptomeria japonica* (Japanese cedar) [10], *Lippia sidoides* (pepper-rosmarin) [11], *Cinnamomum osmophloeum* (cinnamon) [12]*, Syzygium aromaticum* (clove) [13] and *Cymbopogon citratus* (lemongrass) [13, 14] have been reported. EOs have been shown to exert larvicidal effects through at least three different mechanisms: neurotoxicity [15, 16], growth inhibition, and interruption of metabolic pathways [17–19]. However, it should be noted that the relationship between larvicidal activity and the complex chemical composition of a particular EO can be difficult to determine, as interactions among compounds within each EO likely contribute to its killing properties. Further, the combined action of all the components within a particular EO may not only provide a synergistic increase in effectiveness, but likely also prevents the evolution of resistance [18].

Although the efficacy of EOs against larvae of many mosquito species has been demonstrated, there are currently no commercial EO-based larvicides available. While non-toxic to humans and other non-target species at low concentration [20], introduction of large amounts of EO into an aquatic larval environment is likely to disrupt the microbial environment and harm non-target species. In addition, dispersed oils are vulnerable to rapid degradation by UV radiation. The challenge is to deliver essential oils to environments containing mosquito larvae in an efficient, efficacious, and sustainable manner that does not adversely impact the aquatic ecosystem.

*Saccharomyces cerevisiae* (bakers’ yeast) has long been recognized as a viable biocompatible and biodegradable container for a variety of exogenous compounds [21]. Both hydrophobic and hydrophilic food ingredients and pharmaceuticals have been encapsulated into yeast cells for protection, masking, and targeted drug delivery. Incorporating EOs into yeast is accomplished through a simple process using heat and agitation [22]. Once the EO enters the cell, the yeast becomes nonviable. The thick outer envelope of the yeast cell, however, remains intact, and sequesters the oil from the surrounding environment. In this respect *S. cerevisiae* is an ideal field delivery vehicle: it preserves the activity of its payload (EO) while losing the capacity to replicate, and thus to impact aquatic ecosystems. The efficacy of this approach is further facilitated by the fact that mosquito larvae can readily digest *S. cerevisiae* [23]. The cell wall of yeast cells is rich in β-1,3-glucan, a polysaccharide. Larvae have intestinal enzymes specialized for the digestion of β-1,3-glucans and are able to rapidly break down ingested yeast cell membranes [23]. Further, one of the WHO recommended food sources for rearing of mosquito larvae in laboratory settings is *S. cerevisiae* [24].

In this article we describe the development of a novel larvicide consisting of food-grade orange oil (OO) encapsulated into yeast cells. This approach opens a new perspective for the development of a more environmentally friendly larvicide. The defining feature of this formulation is a proprietary washing protocol developed to remove excess EO on the outside of these particles, thereby limiting the amount of EO dispersed into the environment. The microparticles were characterized for EO loading and tested for their efficacy against *Ae. aegypti* larvae.

## Methods

### Larvicide synthesis

Larvicide was synthesized by encapsulation of *Citrus sinensis* EO (orange oil, California origin, Sigma-Aldrich, St. Louis, USA) in *S. cerevisiae* (Red Star fresh baker’s yeast). The encapsulation method is based on an existing process [22, 25], but was optimized to increase encapsulation efficiency for each of the EO tested. A proprietary method (U.S. Provisional Application No 62/752,512) was developed to effectively wash all residual oil from the outside of the microcapsules.

The components used in synthesis of the EO-based larvicide used in this study are orange oil, fresh yeast, and water at ratios of 1:5:16 by weight. Components were placed in a baffled flask and agitated overnight at 40 °C. The solution was then centrifuged, and the supernatant discarded. The larvicide was washed to remove residual oils, then frozen and lyophilized. Larvicide aliquots were rehydrated before use. Similar protocols were used for the encapsulation of essential oils from Australian white cypress, cinnamon leaf, clove bud, lemongrass and thyme.

### Analysis of encapsulated oil

Oil composition analysis and quantification of encapsulated OO was carried out using high performance liquid chromatography (HPLC) on an Agilent 1100 with a temperature-controlled column and UV detector using an Agilent ZORBAX Rx 80 Å C18 column (4.6 × 250 mm with 5 µm particle size). Analysis was performed isocratically at 40 °C using 80% acetonitrile and 20% water mobile phase with flow rate of 1.0 ml/min and UV detection at 214 nm. Full separation of EO components using these conditions were obtained after 14 mins of runtime. Between analyses, the column was washed at 1.5 ml/min with methanol for 4 min, rinsed using a linear gradient from aqueous 1% acetic acid to acetonitrile over 5 min, followed by equilibration at the isocratic analysis ratio for 10 min. For quantification of OO, standard calibration curves for myrcene (Sigma-Aldrich), γ-terpinene (Sigma-Aldrich), and d-limonene (Sigma-Aldrich) at concentrations of 5–1000 mg/l were generated. Retention times for each component were 9.7 min, 11.8 min and 12.3 min, respectively.

Encapsulated oils were extracted from microcapsules by bead milling. Two hundred and fifty mg of rehydrated microcapsules were combined with 300–330 mg of 0.5 mm glass beads and 1.0 ml ethanol in a 15 ml conical vial. The sample was vortexed vigorously for 2 min, centrifuged, and supernatant recovered. A second extraction with another 1.0 ml of ethanol was then performed. The two extracts were combined and filtered through a 0.2 μm PTFE membrane syringe filter (VWR) before HPLC analysis.

### Imaging

Imaging was performed on microcapsules labeled with Nile Red (Sigma-Aldrich). Labeling was performed by adding 0.1 ml of 1 mg/ml Nile Red in DMSO to 1 ml of larvicide suspension (diluted to 10% microcapsules by weight) with shaking at 37 °C for 30 min. Labeled larvicide was washed twice with 10 ml distilled (DI) water after labeling. Wet mounts with labeled cells were prepared using Fluoromount™ (Thermo Fisher Scientific, Waltham, USA) and imaged on a Zeiss AxioObserver microscope equipped with a Hamamatsu Flash4.0v2 sCMOS Camera. Bright field, differential interference contrast (DIC), and fluorescence images were captured.

### Larvicide testing

Larvicidal activity was tested at the University of New Mexico (UNM) and Uniformed Services University of the Health Sciences (USU) using *Ae. aegypti* (Liverpool strain) larvae. Similar experiments were performed at Laboratório de Bioquímica e Fisiologia de Insetos, Instituto Oswaldo Cruz (IOC-Fiocruz) using *Ae. aegypti* (Rockefeller strain) larvae (eggs provided by Laboratório de Fisiologia e Controle de Artrópodes Vetores, IOC-Fiocruz). Eggs were hatched in deionized water (DI) 28 °C with fish food provided *ad libitum*. Once they reached the desired stage, larvae were placed into cups containing 100 ml DI water and measured concentrations of larvicide. Larvicide quantitative bioassays were performed using 1st (L1), 2nd (L2), 3rd (early L3 and L3) and late 3rd/early 4th (L3/L4) instar larvae at three different insectaries. Each cup contains 25 larvae and each dose was replicated on 4 cups for a total of 100 larvae per trial. Larvicide tests were performed at 28 °C. Live and dead larvae were counted after 24 h of larvicide exposure to determine mortality rate at each concentration. All experiments were performed a minimum of 3 times at each site. Mortality curves and lethal doses (LD) values were calculated using the logit generalized linear model implemented in R. All lethal doses are presented with confidence interval at 95% (95% CI).

## Results

### Larvicide analysis

**Fig. 1:**
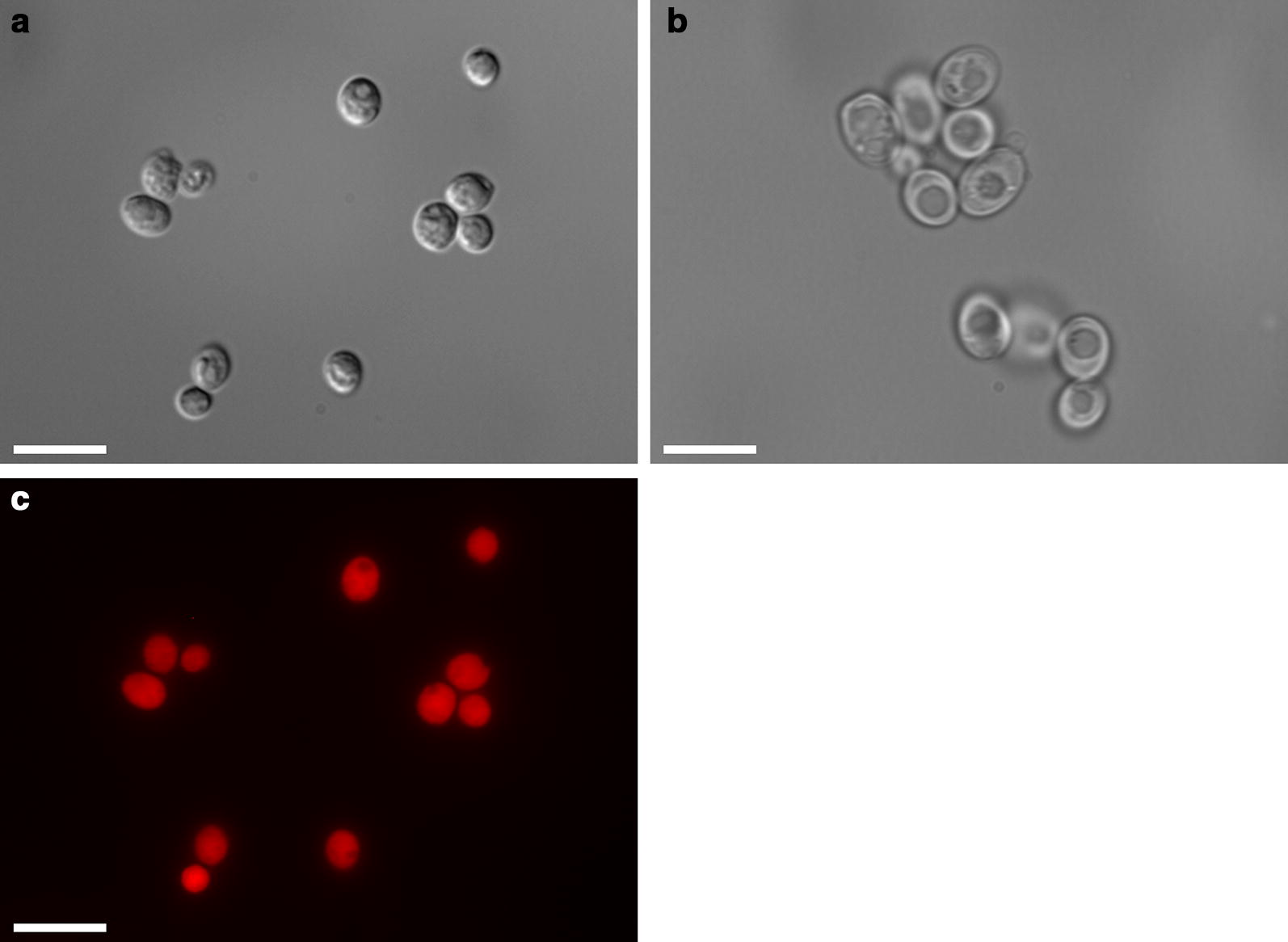
Micrographs of cells with and without encapsulated orange oil (OO). DIC images of cells with (**a**) and without oil (**b**). **c** Fluorescence of cells with oil using the TRITC filter. Cells without oil exhibited no visible fluorescence under these imaging conditions (not shown). *Scale-bars*: 10 μm

Micrographs of larvicide particles labeled with Nile Red show that the individual cells are intact and EO is incorporated within the cells (Fig. 1). Cells without encapsulated oil showed no fluorescence when imaged using a TRITC filter (Ex/Em 557/576) under the same exposure settings as cells with encapsulated oil. Average diameter of the yeast cells was 6.2 μm. Following OO encapsulation, this diameter decreased to an average of 4.4 μm.

**Fig. 2:**
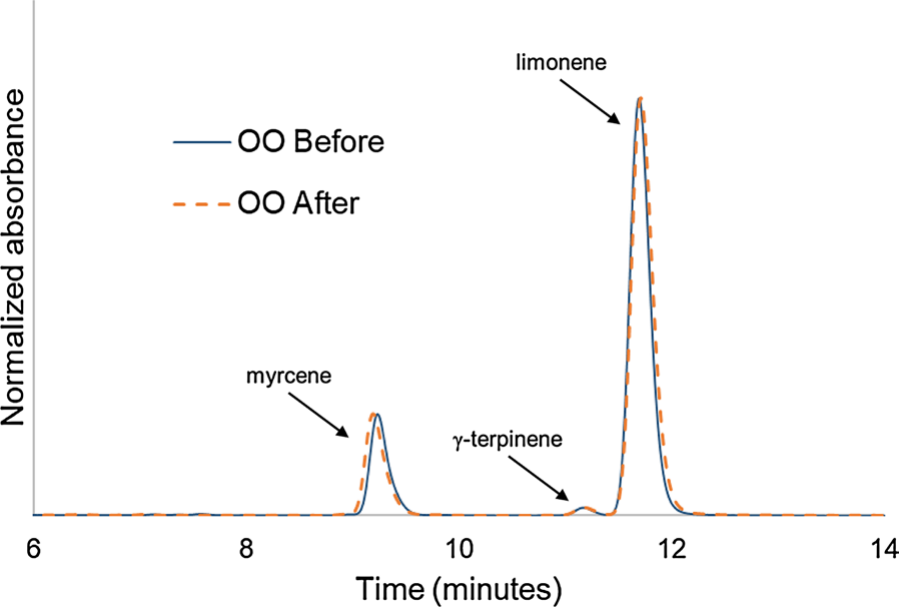
HPLC chromatogram of orange oil (OO) before encapsulation and after extraction from yeast microparticles

Analysis of OO by HPLC before encapsulation yielded a composition of 89.6% d-limonene, 2.4% myrcene, and 1.6% γ-terpinene with 8.2% other minor components, which is consistent with previously reported compositions [26, 27]. After encapsulation, the extracted oil composition was similar: 89.7% d-limonene, 2.2% myrcene, and 1.7% γ-terpinene, as seen in Fig. 2. After encapsulation and washing, but before drying, the oil loading in the larvicide was measured to be from 9.4–10.6 wt%, exhibiting a small amount of batch to batch variability. After lyophilization, the oil loading in the larvicide was 26–30 wt%, which is consistent with the 60–65% reduction in weight observed after the drying process. No significant changes in oil loading, oil composition or larvicidal efficacy were observed with lyophilized samples stored at 4 °C for 6 months.

### Larvicide efficacy

**Fig. 3:**
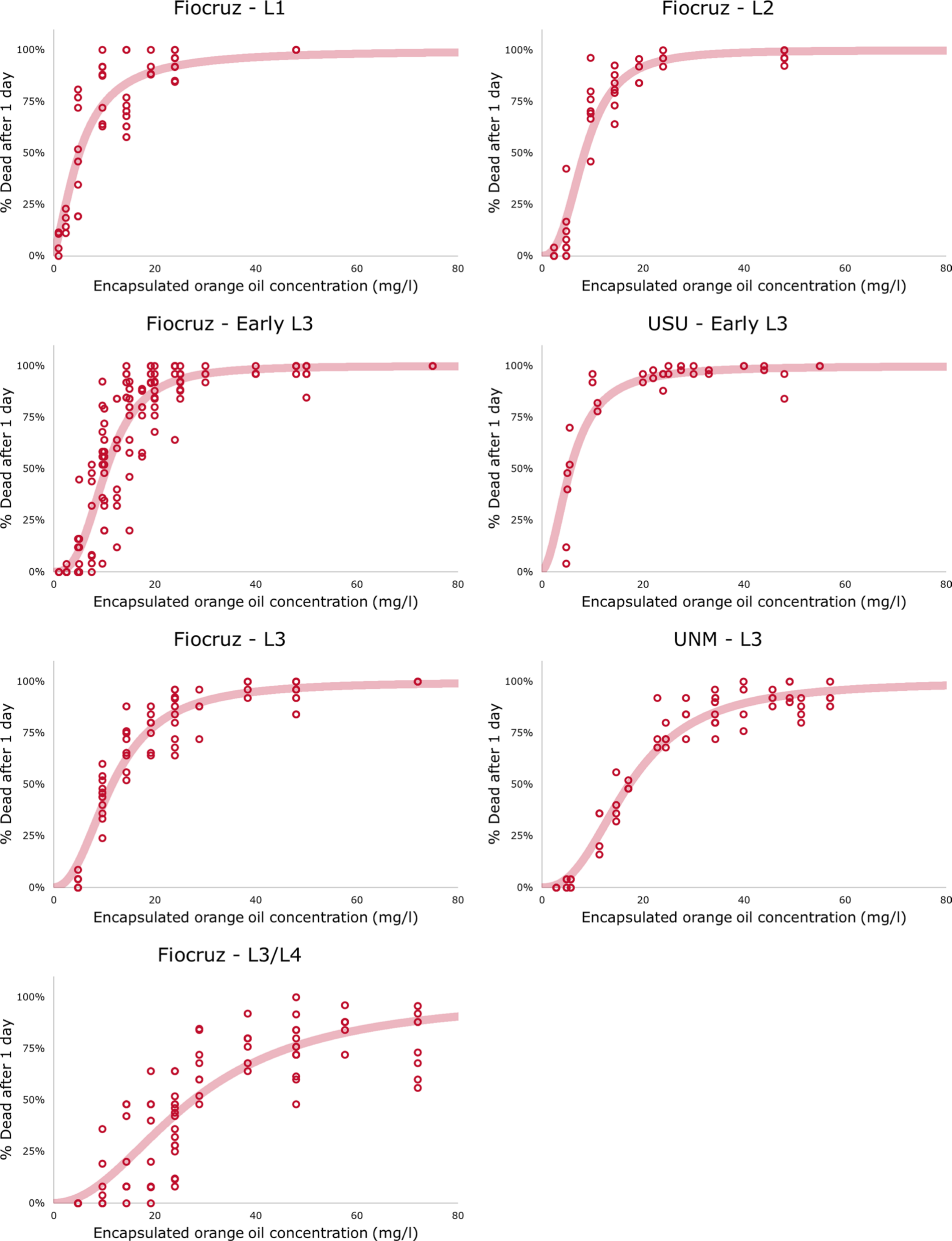
Mortality curves for *Ae. aegypti* larvae. Each point represents one cup with 25 larvae. Efficacy of larvicide was tested at three sites, University of New Mexico (UNM), Uniformed Services University (USU) and Instituto Oswaldo Cruz (IOC-Fiocruz), using larvae at 1st (L1), 2nd (L2), early 3rd (early L3) and late 3rd/early 4th (L3/L4) instar stages. Graphs show larvae mortality following 24 hours of exposure

Mortality at 24 h was observed to be dose dependent at every instar, with lethal dose (LD) as shown in Fig. 3 and Table 1. The LD_50_ and LD_90_ increased sharply as larvae matured, e.g. from 5.1 and 18.9 mg/l, respectively, for L1 larvae to 27.6 and 76.7 mg/l, respectively, for L3/L4 larvae (Table 1). Variation seen between LD_50_ and LD_90_ values may be attributed to the two different laboratory strains of *Ae. aegypti* used at the three study locations. Overall however, these values show a maximum difference of 1.85-fold (LD_50_ for early L3 larvae) between sites (Table 1).

**Table 1:** Computed LD_50_ and LD_90_ values of yeast-encapsulated orange oil against various *Ae. aegypti* larval stages

	Insect stage						
	L1	L2	Early L3	Early L3	L3	L3	L3/L4
Testing institution	Fiocruz	Fiocruz	Fiocruz	USU	Fiocruz	UNM	Fiocruz
LD_50_ (95% CI)	5.1 (4.6–5.6)	8.4 (7.9–9.0)	10.4 (10.1–10.8)	5.6 (5.0–6.3)	11.6 (10.9–12.3)	17.3 (16.3–18.3)	27.6 (26.4–28.8)
LD_90_ (95% CI)	18.9 (16.4–21.7)	17.8 (16.2–19.7)	21.0 (20.0–21.9)	15.3 (13.6–17.2)	28.4 (26.2–30.9)	40.3 (37.2–43.7)	76.7 (69.7–84.3)
*n*	1234	2016	4210	1200	1772	1600	2409

*Notes*: Testing was performed using *Ae. aegypti* Rockefeller strain larvae at Instituto Oswaldo Cruz (IOC-Fiocruz), and on *Ae. aegypti* Liverpool strain at the Uniform Services University (USU) and the University of New Mexico (UNM). Variation in LD values may reflect difference between the two larval strains used. LD values are in mg/l of orange oil. This table is a compilation of all experiments performed at the three sites, with n representing the total number of insects tested at each larval stage. While there were some differences between the LD_50_ and LD_90_ values measured in different research institutes, these measurements fall in the same order of magnitude

Encapsulated Australian white cypress, cinnamon leaf, clove bud, lemongrass and thyme oils were also tested for larvicidal activities. The efficacy of this selection of EOs were previously reported by others [4, 28]. Qualitative microscopy examination of these EO encapsulated cells suggested that similar volumes of encapsulated EO were incorporated into the yeast cells. Larvae were fed encapsulated EO corresponding to 3× the LD_90_ for OO. After 24 h of treatment, mortality of less than 10% was observed with each of these encapsulated EO (data not shown).

## Discussion

The larvicide based on yeast-encapsulated OO was highly active (LD_50_ < 50 mg/l) against all *Ae. aegypti* larval instars (Table 1). LD_50_ is the standard measure for larvicidal activity of natural products such as EO, but effect thresholds can vary [29]. The LD_50_ from bioassays carried out in the three laboratories involved in this study are consistent and ranged from 5.1 to 27.6 mg/l for L1 to L3/L4 larvae respectively. These results confirm our preparation as an active larvicide, with a reproducible mode of delivery [30–32]. This is particularly important due to the high variation in cidal activity of orange oil described in previous studies, including variations in LD_50_ from < 100 mg/l against mosquito larvae [5, 7, 30, 33] to a complete lack of larvicidal activity [6, 8, 34]. In some studies, these differences may be attributed to the methodology used for extraction of the EO, as in the case of larvicidal activities reported for hexane extracts of *Citrus limetta* compared to petroleum ether extracts of the same plant [35].

These results of our bioassays were obtained using two laboratory strains of mosquitoes (Liverpool and Rockefeller) that have been used as reference strains for resistance assays, and as such, may be more sensitive to xenobiotics than insects found in the wild [36]. The efficacy of the OO-yeast particles on larvae progeny (F < 10) from strains of field-caught mosquitoes resistant or susceptible to a variety of commercially used larvicides are currently being evaluated in the laboratory as well as under semi-field conditions at various sites in Brazil. The persistence and impact of this larvicide on non-target organisms, including *Artemia*, *Daphnia* and *Macrobrachium amazonicum* (Amazon River prawn), are also being assessed in the laboratory.

At the onset of our studies, multiple oils were screened for larvicidal activities to determine the best candidate(s) for encapsulation optimization and activity trials. Australian white cypress, cinnamon leaf, clove bud, lemongrass and thyme oils were selected as their primary components differ from those of orange oil. Further, the reported LD_50_ for each of these EO ranges between 0.7–69 mg/l [4]. Their lack of efficacy when administered as encapsulated consumables (data not shown) may be related to different mechanisms of action for these compounds when ingested by larvae, as opposed to being used as contact agents, as in all previous studies [15–19]. Investigation into the mechanism of action after ingestion of encapsulated OO by larvae is ongoing.

Our initial bioassays demonstrated high variability of LD_50_ for all encapsulated EO in a single trial. We hypothesized that presence of EO on the exterior surface of the microcapsules allowed two known properties of these materials to manifest: (i) acting as contact agents which rapidly kill larvae; and (ii) acting as repellents preventing the larvae from eating the microcapsules, even in the absence of other food sources. In response, we developed a washing protocol to remove residual oil for the outside of the yeast particles. Upon implementing this protocol results for larvicidal activity became reproducible both within individual replicates and across different batches. Importantly, results obtained in three different research institutes across the Americas evinced high reproducibility. After our optimized washing process, HPLC analysis of supernatant from concentrated larvicide solution (50 wt% cells ~ 50,000 mg/l encapsulated OO) showed no evidence of d-limonene, myrcene, or γ-terpinene (data not shown).

Interestingly, higher LD_50_ and LD_90_ was observed in L3/L4 larvae. Lower mortality rates in later stage insects may be related to reduced feeding in late L4 mosquito larvae. It has been reported that *Ae. aegypti* larvae, after reaching a critical body mass, stop eating and start preparation to metamorphosis if exposed to stressing factors, like xenobiotics or food with low nutritional value [37]. In this respect, the EO-yeast particles might be inducing pupation in L4 larvae, and that is consistent with the hypothesis that ingestion of the larvicide is an important step in its mechanism of action.

In integrated mosquito control programs, larval source management (LSM) is recognized as successful when it prevents completion of immature mosquito development [38]. The highly anthropophilic *Ae. aegypti* is an urban mosquito living in close proximity to humans and breeding predominantly in human-made containers. These “container-inhabiting” mosquitoes, will breed in almost any aquatic receptacle, from puddles found in flower pots, tires, bottles, gutters to pools of water in communal cisterns and catch basins. Efforts to eliminate *Ae. aegypti* breeding sites are often labor intensive and may require coordination of large numbers of workers, as well as substantial public engagement to sustain community participation in the control efforts [39, 40]. This is further complicated by the fact that eggs of *Ae. aegypti* can withstand desiccation, surviving without water for several months and hatching following the next rainfall [41]. As an alternative, application of organophosphate-based larvicides is costly, harmful to the environment and many strains of *Ae. aegypti* exhibit well documented resistance to these compounds [42]. Similarly, the bioinsecticides *Bacillus thuringiensis israelensis* (Bti) and *Bacillus sphaericus* (Bs) are effective against different species of mosquito larvae under laboratory and environmental settings [42], but their stability is reduced under sunlight and heat exposure [43, 44]. Thus, development of new insecticides effective in tropical climate where mosquito-transmitting diseases are important public health problems, is an urgent need.

The use of insect growth regulators (e.g. pyriproxyfen), entomopathogenic fungi [45] and EO [4] as larvicides have also been reported. However, environmental dissemination of these larvicides continues to be a major obstacle. For pyriproxyfen, this appears to be feasible through auto-dissemination by adult mosquitoes [46, 47]. For EOs, one of the main issues faced for its successful application relates to its’ solubility in an aqueous environment. Ferreira et al. [48], for example, overcame this issue by entrapping orange oil into an *in situ* gelling nanostructured surfactant system, which allowed for improved solubility and contact larvicide activity. To control aphid infestations, Akvetsou et al. [49] encapsulated pennyroyal EO into plasmolyzed yeast cells. These investigators demonstrated a “burst” release of trapped EO from these ruptured yeast cells into the surrounding environment within 30 mins of application. Here, we report on the development of OO laden yeast particles as an ingested larvicide, thereby limiting its impact on non-yeast consuming species. Moreover, the primary components for the synthesis of our larvicide (fresh baker’s yeast and orange oil) are certified food-grade and recognized as environmentally friendly. This technology has the additional advantage of easy adaptability to new formulations depending on the local context e.g. use of alternative essential oils.

## Conclusions

Our novel larvicide based on orange oil encapsulated in yeast was effective against L1–L4 *Ae. aegypti* larvae. Here we have demonstrated an approach that sequesters the cidal compound from the environment and delivers it unmodified to a target species. This approach may provide a solution to the long-standing problem of controlling vector populations with minimal harm to non-target organisms. While the approval and eventual deployment of this larvicide is dependent upon multiple variables, our low-cost product has the potential to function as an initial step in the development of a more environmentally friendly platform technology for the control of multiple vector species.

## Data Availability

Data supporting the conclusions of this article are included within the article. The protocol used for washing of the encapsulated larvicide is proprietary (U.S. Provisional Application No. 62/752,512). The use of yeast-encapsulated EO as a novel larvicide was published as WO2016168837, and is available at https://patentscope.wipo.int/beta/en/detail.jsf?docId=WO2016168837&_cid=B16-K48SGY-75509-1 (Accessed 12/16/2019).

## Abbreviations

DI: deionized
DIC: differential interference contrast
EO: essential oil
IOC-Fiocruz: Instituto Oswaldo Cruz
HPLC: high performance liquid chromatography
LSM: larvae source management
UNM: University of New Mexico
USU: Uniformed Services University
OO: orange oil
wt%: percentage weight
